# *Plasmodium berghei* ANKA: Selection of resistance to piperaquine and lumefantrine in a mouse model

**DOI:** 10.1016/j.exppara.2009.03.010

**Published:** 2009-07

**Authors:** D.M. Kiboi, B.N. Irungu, B. Langat, S. Wittlin, R. Brun, J. Chollet, O. Abiodun, J.K. Nganga, V.C.S. Nyambati, G.M. Rukunga, A. Bell, A. Nzila

**Affiliations:** a Department of Biochemistry, Jomo Kenyatta University of Agriculture and Technology, P.O. Box 62000, 00200 Nairobi, Kenya; b Centre for Traditional Medicine and Drug Research, Kenya Medical Research Institute, P.O. Box 54840, Nairobi 00200, City Square, Kenya; c Institute of Tropical Medicine and Infectious Diseases (INTROMID), Kenya; d Swiss Tropical Institute, Socinstrasse 57, CH-4002 Basel, Switzerland; e Malaria Research Laboratories, University of Ibadan, Ibadan, Nigeria; f Dept. of Microbiology, School of Genetics and Microbiology, Moyne Institute of Preventive Medicine, Trinity College Dublin, Dublin 2, Ireland; g Kenya Medical Research Institute (KEMRI)/Wellcome Trust, Collaborative Research Program, P.O. Box 230-80108, Kilifi, Kenya; h University of Oxford, Nuffield Department of Medicine, John Radcliffe Hospital, Oxford, UK

**Keywords:** Malaria, *Plasmodium berghei*, Drug resistance, Cross-resistance, Lumefantrine, Piperaquine

## Abstract

We have selected piperaquine (PQ) and lumefantrine (LM) resistant *Plasmodium berghei* ANKA parasite lines in mice by drug pressure. Effective doses that reduce parasitaemia by 90% (ED_90_) of PQ and LM against the parent line were 3.52 and 3.93 mg/kg, respectively. After drug pressure (more than 27 passages), the selected parasite lines had PQ and LM resistance indexes (I_90_) [ED_90_ of resistant line/ED_90_ of parent line] of 68.86 and 63.55, respectively. After growing them in the absence of drug for 10 passages and cryo-preserving them at −80 °C for at least 2 months, the resistance phenotypes remained stable. Cross-resistance studies showed that the PQ-resistant line was highly resistant to LM, while the LM-resistant line remained sensitive to PQ. Thus, if the mechanism of resistance is similar in *P. berghei* and *Plasmodium falciparum*, the use of LM (as part of Coartem^®^) should not select for PQ resistance.

## 1 Introduction

Malaria is a global public health priority. The control of malaria is hampered by the rapid selection of parasites resistant to antimalarials. Indeed, there is no single antimalarial in clinical use to which the parasite has not yet developed resistance (Nzila, 2006; White, 2004). Current international strategies for treatment depend on the use of combinations of drugs that include artemisinin compounds. Although this strategy is designed to reduce the chance of resistance emerging, there is considerable concern that this will inevitably happen.

Studies devoted to understanding factors that promote the selection of resistance of *Plasmodium falciparum* to antimalarials have demonstrated that drug elimination profile in the body is one of the key parameters that determine the emergence and selection of resistance (Nzila et al., 2000; Watkins and Mosobo, 1993). When drugs are used in combination, a mismatch between their half-lives can have a substantial impact on the evolution of drug resistance. If one drug is rapidly eliminated, the other drug persists alone and new infections are exposed to sub-therapeutic level of drugs, a fact that promotes the development of resistance (Hastings, 2004).

For instance, the combination of lumefantrine (LM) and artemether (ATM), known as Coartem^®^ has become the first line of treatment of malaria in many African countries, including Kenya (Davis et al., 2005b; Kokwaro et al., 2007; Mutabingwa, 2005; Nosten and White, 2007). Emerging reports indicate that the use of LM in (Coartem^®^) selects for parasites that show an increased tolerance to Coartem^®^ and these parasites select for wild type genotype in, or show increased copy number of *pfmdr*1, a gene associated with chloroquine (CQ) and mefloquine (MFQ) resistance (Dokomajilar et al., 2006; Sisowath et al., 2007, 2005). Thus, there is now concern that resistance to LM will be rapidly selected (Hastings and Ward, 2005; Humphreys et al., 2007; Sisowath et al., 2007).

Piperaquine has been combined with dihydroartemisinin (DHA), the drug known as Artekin^®^. It has undergone successful clinical evaluation in Africa and Asia (Ashley et al., 2004, 2005; Davis et al., 2005a; Denis et al., 2002; Karema et al., 2006; Karunajeewa et al., 2004). Piperaquine has been used as monotherapy for the treatment of malaria infections for several years (in the 80s and early in the 90s) in China. However, when used alone, there was rapid selection of resistance *in vitro*, from 18% in the 1980s to 98% in 1990s (Fan et al., 1998; Yang et al., 1999, 1992; Zhang et al., 1987). This *in vitro*-resistance was followed inevitably by the emergence of *in vivo* resistance (Davis et al., 2005b; Guan et al., 1983; Pang et al., 1989).

In both combinations, the artemisinin derivatives (ATM and DHA) and the main components (PQ and LM) have different pharmacokinetic properties. Indeed, like most artemisinin-based compounds, DHA and ATM are short acting drugs, with a half-lives of less than 2 h (Navaratnam et al., 2000; White et al., 1999). On the other hand, PQ and LM have long half-lives, around 4–6 and 15–20 days, respectively (Ahmed et al., 2008; Hai et al., 2008; Kokwaro et al., 2007; Tarning et al., 2008). Under these circumstances, the selective pressure for resistance would be primarily exerted by the LM and PQ, leading to a rapid selection of PQ and LM resistance when the drug combinations will come into widespread use. In the case of Coartem^®^, a rapid emergence of parasite tolerant to LM have been reported following the use of Coartem^®^ (Dokomajilar et al., 2006; Sisowath et al., 2007, 2005).

Thus, if strategies are to be devised to extend the useful therapeutic lifetime of Coartem^®^ and Artekin^®^, there is a need to understand the mechanisms of PQ and LM resistance. However, to date, there are no well established and characterized PQ- and LM-resistant *P. falciparum* strains, which could be used to study the mechanism of drug resistance.

Here we report the selection of stable LM- and PQ-resistant *Plasmodium berghei* ANKA strains by continuous PQ and LM pressure *in vivo*. We also report the activity of the antimalarial drugs chloroquine (CQ), amodiaquine (AQ), LM and DHA against the backdrop of this LM and PQ resistance. These strains represent valuable tools to study the mechanisms of LM and PQ resistance.

## 2 Materials and methods

### 2.1 Parasites, hosts and test compounds

To select PQ resistance, we used a transgenic ANKA strain of *P. berghei* expressing Green Fluorescent Protein (GFP), resistant to pyrimethamine obtained from the MR4 repository (MRA-865, MR4, ATCC^®^ Manassas, Virginia), while a *P. berghei* ANKA strain expressing GFP-Luciferase fusion, (MRA-868, MR4, ATCC^®^ Manassas, Virginia) obtained from Dr. C.J. Janse of Center of Infectious Diseases Leiden University Medical Center, Netherlands was employed to induce LM resistance. Male, random-bred Swiss albino mice (20 ± 2 g), were each infected intraperitoneally with donor blood containing approximately 2 × 10^7^ parasite red blood cells (PRBC) in 0.2 ml inoculum. However, during the first 4 passages of selection of PQ resistance, female NMRI mice were used and were infected intravenously. Infection was assessed by microscopic estimation of the proportion of infected erythrocytes in Giemsa-stained thin smears made from tail-vein blood.

The animals were housed in experimental room in a standard Macrolon type II cages clearly labeled with experimental details at 22 °C and 60–70% relative humidity and fed on commercial rodent feed and water *ad libitum*.

CQ and AQ were purchased from Sigma Chemical Co. (Poole, UK). DHA, PQ and LM were gifts from Professor Steve Ward, Liverpool School of Tropical Medicine, Liverpool, UK. On the day of administration, the drug was freshly prepared by dissolving it in a vehicle consisting of 70% Tween-80 (*d* = 1.08 g/ml) and 30% ethanol (*d* = 0.81 g/ml) and subsequently diluted 10-fold with double distilled water.

### 2.2 Determination of 50% and 90% effective-dose level (ED_50_ and ED_90_)

Fifty percent and 90% effective doses (ED_50_ and ED_90_, respectively) were measured in a quantitative standard method ‘4-day test’ (4-DT), in which the parasites are exposed to four, daily, drug doses (Peters, 1975), except for the ED_50_ and ED_90_ of the parent strain and that of the line selected at the 4th passage of PQ pressure which were measured using the ‘1-day test’ (1-DT), in which the parasites are exposed to a single drug dose (Vennerstrom et al., 2004). The first 4 passages of PQ pressure were carried out at Swiss Tropical Institute (STI), Basel, Switzerland, using the 1-DT. However, experiments from the 4th passage of PQ pressure and the all LM pressure were carried out at the Kenya Medical Research Institute (KEMRI), Nairobi, Kenya, using the 4-DT. Drugs were administered by oral (po) route on day 1, (24 h post-infection) in the 1-DT or starting on the day 0, (4 h post-infection) and continuing for a total of four daily doses, days 0–3 (24, 48 and 72 h post-infection) in the 4-DT. Parasite count was estimated by microscopic examination of Giemsa-stained thin smears prepared from tail snips on day 3, 72 h post-infection in the 1-DT or on day 4, 96 h post-infection in the 4-DT. Percentage chemosuppression of each dose was then calculated as (A − B)/A] × 100], where A is the mean parasitaemia in the negative control group and B is the parasitaemia in the test group (Tona et al., 2001). ED_50_ and ED_90_ were estimated using a linear regression line.

### 2.3 Procedures for exerting drug-selection pressure and assessing the level of resistance

After inoculation (2 × 10^7^ parasitized red blood cells contained in 0.2 ml inoculums) in 5 mice, on day zero (D0), mice were then orally treated once with the drug at concentration equivalent to ED_99_, 72 h post-infection (D3). Thereafter, parasitaemia was monitored until it reached 2–5%, when a mouse was selected for donation of PRBC to the next naive group of five mice. The parasites were exposed to increasing concentrations of PQ and LM by an ED_99_ factor of one in subsequent passages.

During the first 4 passages of PQ drug pressure, after parasite inoculation (D0), mice (a group of 5) were treated three times with the drug at concentration equivalent to ED_99_. The first treatment was carried out 72 h post-infection (D3). The second and third treatment followed on D6 (or 7) and 10. Drugs were administered orally in a volume of 0.01 ml per gram mouse. After the third treatment (D10), parasitaemias were monitored until they reached ⩾2% when a mouse was selected for donation of PRBC to the next naive group of five mice and subsequent steps were carried out as mentioned in the previous paragraph.

The level of resistance was evaluated at different intervals by measurement of ED_50_ and ED_90_ in the standard 4-DT or 1-DT which permits the calculation of an ‘index of resistance’, I_50_ and I_90_ (defined as the ratio of the ED_50_ or ED_90_ of the resistant line to that of the sensitive, parent line).

The I_90_ values were grouped into four categories, based on previous work (Merkli and Richle, 1980): (1) I_90_ = 1.0, sensitive, (2) I_90_ = 1.01–10.0, slight resistance, (3) I_90_ = 10.01–100.0, moderate resistance and (4) I_90_ > 100.0, high resistance.

### 2.4 Stability study

The stability of PQ and LM resistant line was evaluated by measuring drug responses after (i) making 10 drug free passages followed by measurement of ED_90_; (ii) freeze-thawing of parasites from −80 °C followed by measurement of ED_90_. Stable resistance was defined as the maintenance of the resistance phenotype when drug-selection pressure was removed for at least 10 passages in mice (Gervais et al., 1999).

### 2.5 Cross-resistance studies

The activity of CQ, AQ, LM and DHA against both drug sensitive and resistant lines (after 10 drug free passages) was assessed in the 4-DT. I_90_ was computed as the ratio of the ED_90_ of the resistant line to that of the sensitive, parent line. Cross-resistance was classified into three categories as previously described (Li, 1985; Li et al., 1985): I_90_ ⩽1.00 sensitive, I_90_ of 1.01–5.00 as slight cross-resistance, I_90_ of above 5.01 as high cross-resistance. Statistical analyses were carried out using the Student *t*-test (Minitab Inc. software, State College, PA, USA).

## 3 Results

The ED_50_ and ED_90_ of PQ against the parent line were 1.30 and 3.52 mg/kg, respectively. After 4 passages under PQ selective pressure (a total of 15 treatments), the PQ ED_50_ and ED_90_ increased to 160.28 and 262.59 mg/kg, respectively, yielding I_50_ of 123.29 and I_90_ of 74.70 (Table 1). However, after cryopreservation and revival of the parasite, this resistance decreased, with ED_50_ and ED_90_ of 7.50 and 21.90 mg/kg, respectively (Table 1).

**Fig. 1:**
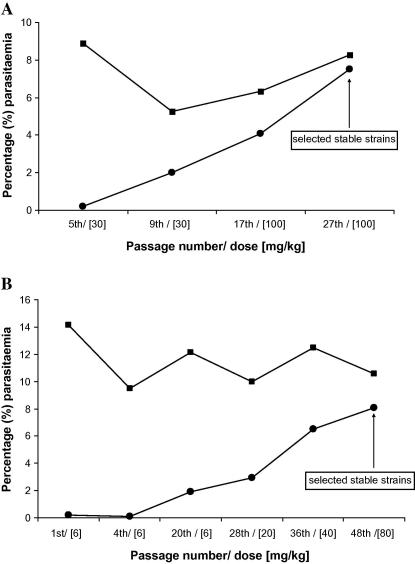
Development of parasitaemia in the treated (●) and untreated mice (■) at different levels during the selection of piperaquine (A) and lumefantrine (B) resistant *Plasmodium berghei* GFP (for PQ) and GFP-Luciferase (for LM) ANKA strains in mice. Parasitaemias were assessed after 4 days post-infection (in both control and treated groups) and mice were treated using a 4-day test (4-DT, see Materials and methods).

When exposed to further 23 passages (27th passage) of selection pressure, parasites regained the resistant phenotype and reached a high level of resistance with I_50_ and I_90_ of 129.29 and 68.86, respectively (Table 1). Fig. 1A shows the changing response of the *P. berghei* ANKA to PQ in the course of PQ drug pressure. After the 5th passage under PQ pressure, a dose of 30 mg/kg (>8 times higher the ED_90_ of the parent strain) suppressed the bulk of parasitaemia, indeed treated mice had parasitaemia of 0.22% only, compared to almost 9% of the untreated group. Thereafter, PQ resistance arose quite rapidly from the 9th passage. Infected mice treated with 30 mg/kg could yield parasitaemia of 2% at the 9th passage, and at 17th passage, parasitaemia reached 4% after a higher dose, 100 mg/kg. The continuous PQ pressure to 27th passage allowed the selection of parasite lines that reached 7.5% (parasitaemia almost as high as the control [8.26%]), after treated mice with 100 mg/kg, a clear indication of the rise in resistance. This resistant phenotype was stable and these parasite lines were scored as PQ-resistant strains (Table 1 and Fig. 1A).

Results pertaining to selection of LM resistance are summarised in Table 2. ED_50_ and ED_90_ of the parent line were 1.67 and 3.93 mg/kg, respectively. The continuous LM pressure after 48 consecutive passages led to the selection of a parasite line yielding ED_50_ of 140.15 and ED_90_ of 249.75 corresponding to an I_50_ and I_90_ of 83.92 and 63.55, respectively. Such values of the I_50/90_ indicate that the parasite developed resistance to the drug.

Like with PQ, the first 5 passages were not associated with increase in LM resistance. At the 5th passage, a dose 6 mg/kg allowed a parasitaemia of 0.08% only (Fig. 1B) However, at the 20th passage, at the same dose (6 mg/kg), parasite grew and reached 2% parasitaemia, a clear indication of emergence of resistance. This resistance increased further with the number of passages. At 28th, a dose of 10 mg/kg did not prevent parasitaemia to reach 4.1%, and at 36th, a higher parasitaemia of 6.5% was reached at a dose of 40 mg/kg. The higher level of resistance was observed at 48th passage, with mice harboring parasitaemia of 8% when treated with 80 mg/kg. During all 48 passages, parasitaemias in the untreated controls remained steady, ranging between 9% and 12%, a confirmation of dramatic rise in LM resistance (Fig. 1B). At this point (48th passage), a stable LM-resistant strain was selected (Fig. 1B, Table 2).

These high values of PQ and LM resistance indices led us to test the stability of the resistant phenotypes. We maintained these two resistant strains in absence of the drug pressure for 10 passages (at least 2 months) and then assessed *in vivo* activity of the drugs. The resulting PQ I_50_ and I_90_ remained high, with values of 147.28 and 83.80, respectively, and those of the LM were 79.74 for I_50_ and 65.19 for I_90_, a clear indication of the stability of the resistant phenotype. To further check this stability, we cryo-preserved these parasite lines for 2 and 4 months for LM and PQ, respectively. Upon revival, the analysis of the drug activity showed PQ I_50_ and I_90_ indexes of 84.64 and 63.39, respectively, and those for LM as 69.66 and 52.06, respectively. These values are slightly lower than those obtained before cryopreservation. However, they remained high, 50–80 times higher than those of the parent lines, a further indication of the stability of the phenotype (Tables 1 and 2).

We also tested the extent to which resistance to PQ and LM affect the activity of other antimalarial drugs, a phenomenon known as cross-resistance, and the results are summarized in Tables 3 and 4. Against the PQ-resistant line, the activity of AQ, CQ and DHA decreased significantly by a factor of 3–7 (*p* < 0.01 at least) (Table 3), an indication of the existence of slight cross-resistance of PQ with AQ, CQ and DHA. Surprisingly, the highest level of cross-resistance was recorded with LM, with its activity decreasing 97-fold against this PQ-resistant parasite line (*p* < 0.0001).

Overall, the LM-resistant parasite line retained relative susceptibility to the 4-aminoquinolines, AQ and PQ (Table 4). Indeed, AQ activity did not change (I_90_ of 1.06), and more interestingly, this parasite line remained susceptible to the bisquinoline PQ (I_90_ of 0.91). However, a significant decrease in activity was observed with the aminoquinoline CQ, with an I_90_ of 1.62 (*p* < 0.0001), and the endoperoxide DHA, with an I_90_ of 5.36 (*p* < 0.05). Thus the selection of LM resistance is associated with a decrease in CQ and DHA activity and the retention of AQ and PQ susceptibility.

## 4 Discussion

Our study shows that PQ and LM resistance in *P. berghei* ANKA can be selected within 18 months of continuous drug pressure. To the best of our knowledge, this is the first report of the selection of stable PQ- and LM-resistant strains in murine malaria following drug pressure. A PQ-resistant *P. berghei* strain had been selected in 5 months of selection pressure, but when the drug was removed, the strain reversed to sensitive phenotype (Li, 1985; Li et al., 1985), and a stable phenotype was observed only after mouse–mosquito–mouse passages (Li et al., 1985).

Two approaches by other laboratories have been used to select resistant murine malaria parasites: the 2% relapse technique (2% RT) in which a single and high drug dose is administered at the time of each passage (Peters and Robinson, 1999) and the serial technique (ST), in which drug dose is gradually increased after each passage (Peters, 1999; Peters and Robinson, 1999).

Using 2% RT, a number of phenotypes stably resistant to pyronaridine, amodiaquine, atovaquone and tafenoquine have been selected in *P. berghei* (Peters and Robinson, 1992, 1999, 2000; Peters et al., 2003) and tafenoquine in *Plasmodium yoelii* and *Plasmodium chabaudi* (Peters et al., 2003). However, using this method, stable resistance to sulfadoxine/pyrimethamine in *P. berghei* and artemisinin in *P. yoelii* could not be selected (Peters, 1999; Peters and Robinson, 1999). On the other hand, the ST approach has allowed the establishment of strains stably resistant to various antimalarials, including atovaquone in *P. berghei* (Syafruddin et al., 1999) and mefloquine in *P. chabaudi*, (Cravo et al., 2003), artemisinin in *P. chabaudi* (Afonso et al., 2006), halofantrine in *P. yoelii* (Singh and Puri, 2000) and arteether in *P. vinckei* (Puri and Chandra, 2006). Though failure to select stable resistance to piperaquine, chloroquine and primaquine in *P. berghei* has been reported (Li, 1985; Peters, 1999; Peters et al., 2003), overall, the ST approach has proven to be more efficient to select for stably resistant strains than 2% RT (Afonso et al., 2006; Cravo et al., 2003; Puri and Chandra, 2006). Using this approach, we have, for the first time, successfully established stable PQ- and LM-resistant *P. berghei* strains within 12–18 months of drug pressure.

Interestingly, after cryopreservation of both PQ and LM-resistant strains, a decrease in ED_50/90_ was recorded upon revival of the strains. This is common, it indicates that some of the mechanisms of resistance are the result of epigenetic changes such as gene amplification, protein over expression and protein modifications. However, if resistance is well established, the degree of ED_50/90_ decrease is small, the strains remained resistant to the drugs, as our data show.

Evaluation of cross-resistance patterns revealed that PQ and AQ retain potency against the LM resistant parasite line. LM is an arylaminoalcohol closely related to mefloquine (MQ), halofantrine and pyronaridine (Schlitzer, 2008). PQ, AQ and CQ are 4-aminoquinoline derivatives, and are likely to share a similar mechanism of action (Raynes, 1999). Resistance to CQ and AQ in *P. falciparum* is reported to be inversely correlated with resistance to arylaminoalcohols (Duraisingh and Cowman, 2005), and the selection of the resistance to arylamino-alcohol MQ results in an increase in CQ sensitivity (Cowman et al., 1994; Peel et al., 1993). In our experiments, LM resistance was not associated with a decrease in PQ efficacy. Similarly, the efficacy of the 4-aminoquinolines AQ and CQ did not change or only slightly decreased against the LM-resistant strain. Assuming that the mechanism of LM resistance is similar in *P. falciparum* and *P. berghei*, these results would suggest a high efficacy of PQ against LM-resistant strains in *P. falciparum*.

It is very interesting to note the activity of LM against PQ-resistant line decreased by 97-fold, a rate which is even higher than its activity against the LM-resistant parasite line selected after 2 years of LM pressure (I_90_ of 64). Thus, the selection of PQ resistance is associated with a higher level of LM resistance, while, as discussed earlier, the selection of LM resistance is associated with PQ susceptibility. This demonstrates that two different LM-resistance phenotypes exist. The first phenotype is associated with PQ resistance, while the second is associated with PQ susceptibility. Assuming that the same pattern prevails in *P. falciparum*, the use of either drug could be associated with resistance or susceptibility to the other. For instance, currently, Coartem^®^ is being used to treat malaria, thus the selection of resistance to LM could be associated with susceptibility to PQ (component of Artekin^®^). While if Artekin^®^ is first used, resistance to this drug may render Coartem^®^ ineffective.

Our data show significant 3- and 5-fold decreases in dihydroartemisinin activity against PQ- and LM-resistant strains, respectively, an indication of the existence of a slight cross-resistance between LM and artemisinin, and PQ and artemisinin, in *P. berghei*. In *P. falciparum*, resistance to the arylamino-alcohol mefloquine, as the result of the increase copy number of *pfmdr*1, is associated with a decrease in activity of artemisinin derivatives (Nelson et al., 2005; Pickard et al., 2003; Price et al., 1999), thus a similar phenomenon may prevail in *P. berghei* with the arylamino-alcohol LM. We report cross-resistance between PQ and artemisinin in *P. berghei*, in agreement with previous work (Li, 1985; Li et al., 1985).

Thus, if the mechanism of LM and PQ resistance is similar in *P. berghei* and *P. falciparum*, the selection of LM and PQ resistance would be associated with a reduced artemisinin derivative efficacy, compromising the potential of artemisinin-based combinations. Consequently, there is an urgent need to clarify the mechanism of LM and PQ resistance and establish the extent of cross-resistance between these important antimalarials. The existence of cross-resistances to chemically and mechanistically unrelated drugs suggests the likely involvement of changes in drug accumulation, i.e. a ‘multi-drug resistance’ phenotype.

The LM and PQ resistant parasite lines have been selected so as to study the mechanism of drug resistance in *P. berghei* and use this information as platform to explore the resistance mechanism in *P. falciparum*. In the latter species, reports indicate that the use of LM + ATM (Coartem^®^) selects for 2 haplotypes at 86Y-184Y-1246Y and 86Y-184F-1246D of *pfmdr*1, a gene associated with changes in susceptibility to chloroquine (Dokomajilar et al., 2006; Humphreys et al., 2007; Sisowath et al., 2007, 2005). The copy number of *pfmdr*1 has also been reported to increase with the use of LM in field isolates in Thailand (Price et al., 2006), and a decrease in copy number was found to heighten *in vitro* lumefantrine susceptibility in laboratory selected parasites (Duraisingh and Cowman, 2005; Sidhu et al., 2006). These observations indicate that *pfmdr*1 will likely contribute to LM resistance, but the full definition of the mechanism of resistance remains to be elucidated since overall, Coartem^®^ retains sensitivity against CQ-resistant isolates. In *P. berghei* and *P. chabaudi*, amplification of the *pfmdr*1 orthologue is associated with mefloquine resistance (Carlton et al., 2001) as in *P. falciparum* (Cowman et al., 1994; Peel, 2001; Sidhu et al., 2005). Thus, *pfmdr*1 could also be involved in LM resistance in *P. berghei*.

PQ is a bis-chloroquine derivative (Davis et al., 2005a; Raynes, 1999). Thus one could expect that PQ and CQ would share the same mode of action and perhaps a similar mechanism of resistance. However, PQ remains active against CQ-resistant isolates (Basco and Ringwald, 2003), clearly indicating that though PQ is closely related to CQ, these two drugs have different mechanisms of resistance. To date, no gene or candidate gene has been associated with PQ resistance in *P. falciparum*. Thus, further analysis of this PQ resistant *P. berghei* line could provide insight into the mechanism of PQ resistance.

However, we are aware that mechanism of resistance in *P. falciparum* and murine *Plasmodium* species may be different. For instance, the mechanisms of resistance to CQ are different in *P. falciparum* and in murine malaria and there is still a debate whether those of artemisinin derivatives will be similar (Afonso et al., 2006; Carlton et al., 2001; Hunt et al., 2007, 2004a,b). However, for drugs such as mefloquine, antifolates, and atovaquone, similar mechanisms of resistance have been reported (Carlton et al., 2001). Thus, the use of murine malaria could provide critical information on the mechanisms of resistance to PQ and LM.

In summary, we have selected LM and PQ resistant lines of *P. berghei*. The stability of this phenotype indicates that mechanisms that underlie it are coded into the cell genome. Amplification of *pfmdr*1 has been associated with resistance to mefloquine, an amino-alcohol (Carlton et al., 2001). We hypothesise that the same could prevail in LM, which is also an amino-alcohol. Studies are underway to explore the mechanisms of resistance to LM and PQ.

## Figures and Tables

**Table 1:** Selection of piperaquine resistance in *Plasmodium berghei* GFP ANKA strain using serial technique. Data are presented as effective doses that reduce parasitaemia by 50% and 90% (ED_50_, ED_90_) and as 50% and 90% indexes of resistance (I_50_ and I_90_, defined as the ratio of the ED_50_ or ED_90_ of the resistant line to that of the parent strain).

Passage no.	ED_50_ (mg/kg)	(I_50_)	ED_90_ (mg/kg)	(I_90_)
Parent	1.30	1	3.52	1
4th	160.28	123.29	262.59	74.70
(6 months cryopreservation) (5th passage)	7.50	5.77	21.90	6.22
9th	21.40	16.46	64.50	18.32
17th	122.00	93.85	194.00	55.11
27th	168.08	129.29	242.38	68.86

*Drug free passages*				
5th	185.27	142.52	283.71	80.60
10th	191.46	147.28	294.98	83.80
27th passage line after 4 months cryopreservation	110.03	84.64	223.15	63.39

**Table 2:** Selection of lumefantrine resistance in *Plasmodium berghei* GFP-Luciferase ANKA strain using serial technique. Data are presented as effective doses that reduce parasitaemia by 50% and 90% (ED_50_, ED_90_) and as 50% and 90% indexes of resistance (I_50_ and I_90_, defined as the ratio of the ED_50_ or ED_90_ of the resistant line to that of the parent strain).

Passage no.	ED_50_ (mg/kg)	(I_50_)	ED_90_ (mg/kg)	(I_90_)
Parent	1.67	1	3.93	1
4th	1.34	0.80	3.49	0.89
12th	1.58	0.95	3.25	0.83
20th	2.96	1.77	5.25	1.34
28th	9.76	5.84	25.50	6.49
36th	42.50	25.33	69.70	17.74
48th	140.15	83.92	249.75	63.55

*Drug free passages*				
10th	133.17	79.74	256.21	65.19
48th line after 2 months cryopreservation	116.34	69.66	204.58	52.06

**Table 3:** Response of piperaquine resistant *Plasmodium berghei* GFP ANKA line to amodiaquine (AQ), chloroquine (CQ), lumefantrine (LM) and dihydroartemisinin (DHA). Results are presented as effective doses that reduce parasitaemia by 90% (ED_90_) and as 90% indexes of resistance (I_90_, defined as the ratio of the ED_90_ of the resistant line to that of the sensitive, parent strain).

Antimalarial drug	ED_90_⁎ (mg/kg)		Index of resistance (I_90_)
	Parent strain	Resistant line	
AQ	3.72	13.48§	3.62
LM	2.52	245.06¶	97.25
CQ	3.57	26.24‡	7.35
DHA	4.08	12.06§	2.96

⁎ Differences between parent and resistant lines were significant according to Student’s *t*-test.

‡ *p* < 0.01.

§ *p* < 0.001.

¶ *p* < 0.0001.

**Table 4:** Response of lumefantrine resistant *Plasmodium berghei* GFP-Luciferase ANKA line to amodiaquine (AQ), piperaquine (PQ), chloroquine (CQ) and dihydroartemisinin (DHA). Results are presented as effective doses that reduce parasitaemia by 90% (ED_90_) and as 90% indexes of resistance (I_90_, defined as the ratio of the ED_90_ of the resistant line to that of the sensitive, parent strain).

Antimalarial drug	ED_90_⁎(mg/kg)		Index of resistance (I_90_)
	Parent strain	Resistant line	
AQ	4.29	4.53†	1.06
PQ	3.70	3.37†	0.91
CQ	4.47	7.22¶	1.62
DHA	6.69	35.86‡	5.36

⁎ Differences between parent and resistant lines were analyzed by Student’s *t*-test.

† *p* > 0.05 (insignificant).

‡ Significant at *p* < 0.05.

¶ Significant at *p* < 0.0001.
